# The Acyl-CoA Specificity of Human Lysine Acetyltransferase KAT2A

**DOI:** 10.1021/acs.biochem.2c00308

**Published:** 2022-08-22

**Authors:** Ananya Anmangandla, Yuxiang Ren, Qin Fu, Sheng Zhang, Hening Lin

**Affiliations:** † Department of Chemistry and Chemical Biology, Cornell University, Ithaca, New York 14853, United States; ‡ Proteomics and Metabolomics Facility, Cornell University, Ithaca, New York 14853, United States; § Howard Hughes Medical Institute and Department of Chemistry and Chemical Biology, Cornell University, Ithaca, New York 14853, United States

## Abstract

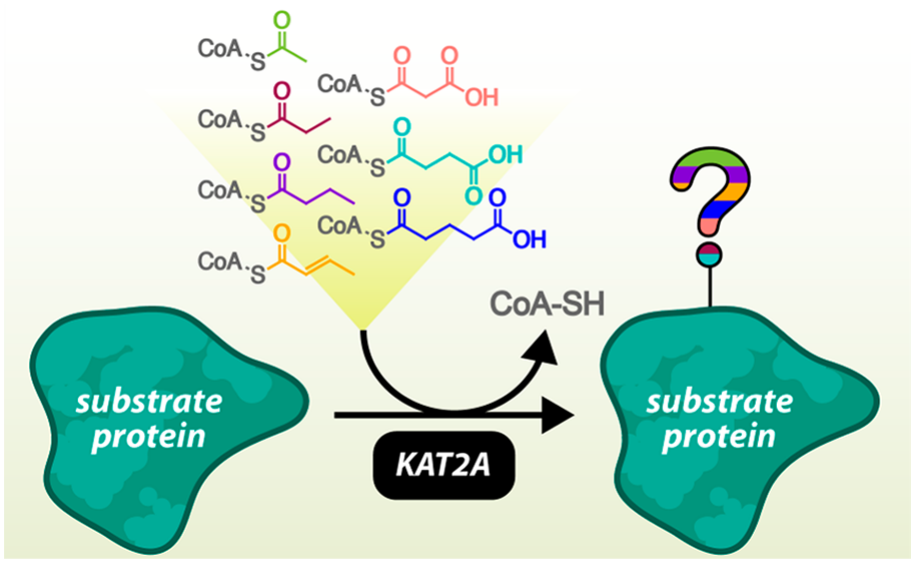

Protein post-translational modifications serve to regulate a broad range of cellular functions including signal transduction, transcription, and metabolism. Protein lysine residues undergo many post-translational acylations and are regulated by a range of enzymes, such as histone acetyl transferases (HATs) and histone deacetylases (HDACs). KAT2A, well characterized as a lysine acetyltransferase for both histone and nonhistone substrates, has been reported to tolerate additional acyl-CoA substrates, such as succinyl-CoA, and shows nonacetyl transferase activity in specific biological contexts. In this work, we investigate the acyl-CoA substrate preference of KAT2A and attempt to determine whether and to what extent additional acyl-CoA substrates may be utilized by KAT2A in a cellular context. We show that while KAT2A can bind and utilize malonyl-CoA, its activity with succinyl-CoA or glutaryl-CoA is very weak, and acetylation is still the most efficient activity for KAT2A *in vitro* and in cells.

## Introduction

Lysine post-translational modifications (PTMs) have been described for several decades, and much effort has been made to characterize regulation and functions of lysine acetylation, methylation, and ubiquitination.^1^ Recently, many additional protein lysine acylations have been reported. These include lysine butyrylation,^2^ crotonylation,^3^ propionylation,^2^ malonylation,^4^ succinylation,^5,6^ glutarylation,^7^ lactylation,^8^ and benzoylation,^9^ among others. However, the functions of most of these recently identified lysine acylations have not been extensively characterized and the readers, writers, and erasers for these modifications have not been comprehensively determined.

Lysine acetyl transferase KAT2A is annotated as part of the HAT family and has been shown to bind acetyl-CoA and acetylate multiple histone lysine residues.^10,11^ In addition to its well-known histone targets, KAT2A can acetylate several nonhistone targets including the cell-division cycle (CDC)-6 protein to regulate cell cycle progression,^12^ CCAAT enhancer binding protein beta (C/EBPβ) to increase its transcriptional activation,^13^ and polo-like kinase 4 (PLK4) to prevent centrosome amplification.^14^

However, additional activities for KAT2A, including succinylation and glutarylation of histone substrates, have been reported and even annotated in the UniProt database.^15,16^ Specifically, it was reported that KAT2A succinylates lysine 79 of histone H3 in complex with α-ketoglutarate dehydrogenase (α-KGDH). α-KGDH, responsible for succinyl coenzyme A (succinyl-CoA) generation in the nucleus, binds KAT2A and facilitates its histone succinyltransferase activity via *in situ* generation of the succinyl-CoA substrate. Similarly, it is reported that KAT2A is responsible for glutarylation of lysine 41 on histone H4 with the α-ketoadipate dehydrogenase (α-KADH) complex.^16^ α-KADH shares the same E2 and E3 components as the α-KGDH complex, and KAT2A binds to the unique E1 components of each complex to carry out the corresponding activities.^17^

Given these reported new acyltransferase activities of KAT2A, we became interested in comparing the activity of KAT2A on different acyl-CoA substrates. We reasoned that such knowledge would be important for understanding the cellular function of KAT2A and whether the function is through acetylation or other acylation activities. Surprisingly, our data suggest that the reported succinyltransferase activity of KAT2A is very weak in comparison to its enzymatic acetyltransferase activity and most of the succinylation occurs nonenzymatically. The malonyltransferase activity of KAT2A *in vitro* is detectable, but our data support that the major activity of KAT2A *in vitro* and in cells is still acetylation.

## Materials and Methods

### Reagents

All reagents and solvents were analytical grade and purchased from commercial vendors. H3.1 was purchased from NEB (M2503S), and isolated calf thymus histone was purchased from Sigma (10223565001). KAT2A full length was purchased from Cayman Chemical (10782). CoAs was purchased from Santa Cruz [acetyl-CoA sodium salt (sc-210745A), crotonyl-CoA trilithium salt (sc-300396), glutaryl-CoA lithium salt (sc-215074), malonyl-CoA lithium salt (sc-215286B), propionyl-CoA lithium salt (sc-215475), succinyl-CoA sodium salt (sc-215917)] or Cayman Chemical [butyryl-CoA sodium salt (27865)]. Peptides were purchased from Biomatik. Acetyl, malonyl, and succinyl-lysine antibodies were purchased from PTM Biolabs (PTM-101, PTM-901, PTM-401, respectively). KAT2A rabbit mAb antibody was purchased from Cell Signaling Technology (C26A10).

### HPLC Conditions

The analytical HPLC used to monitor the enzymatic reactions of KAT2A was Shimadzu HPLC LC20-AD with a Kinetix 5 μm EVO C18 100 Å column (100 mm × 4.60 mm, 5 μm), at 215 and 280 nm. Solvents used for the analytical HPLC were water with 0.1% HPLC-grade trifluoroacetic acid and acetonitrile with 0.1% HPLC-grade trifluoroacetic acid.

### Cloning, Expression, and Purification of KAT2A

Human KAT2A catalytic domain (497–662) was cloned into the pET28a vector with an N-terminal His tag using the *Eco*RI and XhoI sites. The sequenced plasmid was transformed into BL21(DE3) chemically competent *E. coli.* Then, 4 L of LB broth with 50 μg/mL of Kanamycin was inoculated with an overnight starter grown at 37 °C. Cultures were grown at 200 rpm and 37 °C for ∼4 h until the OD_600_ reached 0.8. Then, IPTG was added to 0.5 mM, and the cells were incubated at 16 °C overnight to allow protein expression. Cells were harvested by centrifugation at 6000*g*. Cell pellets were frozen at −80 °C or immediately used for purification. Pellets were resuspended in lysis buffer (50 mM Tris pH 8.0, 500 mM NaCl, 0.5 mg/mL lysozyme, 1 mM PMSF, and Pierce universal nuclease). Following a 30 min incubation, cells were sonicated on ice for 4 min total at 60% amplitude. Lysate was clarified at 4 °C and 30 000*g* for 35 min. Clarified lysate was loaded onto Ni-NTA resin, washed with 50 mL wash buffer (50 mM Tris pH 8.0, 500 mM NaCl, 20 mM imidazole), and eluted with elution buffer (50 mM Tris pH 8, 500 mM NaCl, 200 mM imidazole). Crude KAT2A was concentrated using a 10 kDa MWCO Amicon filter and loaded onto a Superdex 75 gel filtration column equilibrated with a storage buffer (25 mM HEPES pH 8.0, 200 mM NaCl) on an ÄKTA pure FPLC system. Fractions containing KAT2A (497–662) were pooled, concentrated, flash-frozen in liquid nitrogen, and stored at −80 °C for future use.

### KAT2A Peptide Activity Assay and acyl-CoA Substrate Screen

A reaction buffer (25 mM NH_4_HCO_3_) containing 100 μM H3K9 peptide (sequence KQTARKSTGGKWW) or H3K79 peptide (sequence EIAQDFKTDLRFQWW) was prepared. Stock solutions of acyl-CoAs (acetyl-CoA, butyryl-CoA, crotonyl-CoA, glutaryl-CoA, malonyl-CoA, propionyl-CoA, and succinyl-CoA) were prepared in water. Reactions were prepared with or without the 500 nM purified KAT2A catalytic domain (497–662). For initial H3K9 peptide activity assays, samples were incubated with 0, 5, 20, 100, and 400 μM of acyl-CoA. For H3K79 peptide activity assays, samples were incubated with 100 μM peptide and 400 μM CoA. For the acyl-CoA screen with H3K9 peptide, 300 μM CoA was added to initiate the reaction. All samples were incubated at 37 °C for 30 min and quenched with an equal volume of acetonitrile. After vortexing and centrifuging at 17 000*g* for 5 min to remove the precipitated enzyme, the supernatant was loaded to HPLC with a Kinetex EVO C18 column (100 × 4.60 mm, 5 μM, 100 Å) for analysis. All experiments were performed at least in duplicate.

### KAT2A Recombinant Histone Activity Assay

Commercially purchased recombinant histone H3.1 (1 μg) or calf thymus (5 μg) was added to the reaction buffer (25 mM Tris pH 8.0, 200 mM NaCl) with or without 500 nM KAT2A (497–662) or 125 nM commercially purchased FL KAT2A. Acetyl-, succinyl-, or malonyl-CoA was added to appropriate samples to initiate the reaction at final concentrations of 0, 5, or 50 μM. Reactions were incubated at 37 °C for 20 min, quenched with 6× SDS denaturing load dye (60 mM Tris pH 6.8, 12% SDS, 0.06% bromophenol blue, 50% glycerol, 600 mM DTT), and boiled for 5 min before loading onto an SDS-PAGE gel for analysis. Lysine acetylation, succinylation, and malonylation were determined by Western blot using PTM Biolabs PTM-101, 401, and 901 antibodies, respectively. Membranes were stained with Coomassie Blue to assess loading.

### Determining Changes Acetylation, Succinylation, and Malonylation with KAT2A Knockdown

For transient knockdown of KAT2A, lentivirus was produced by PEI transfecting HEK 293T cells with psPAX packaging vector, VSV-G envelope vector, and KAT2A Sigma MISSION shRNAs (sh1, TRCN0000038883; sh2, TRCN0000307319; sh3, TRCN0000294334; sh4, TRCN0000286981). The medium was replaced with fresh medium containing 1 mM sodium pyruvate. Medium containing lentiviral particles was harvested every 24 h for 72 h. HEK 293T cells were seeded into a six-well plate 18 h prior to transduction to 30% confluency. Cells were transduced with virus particles in media supplemented with 6 μg/mL Polybrene. The medium was replaced with fresh medium without Polybrene after 12 h, and cells were harvested 72 h post transduction for maximum knockdown efficiency. SiRNA knockdown was done following standard transfection protocols with Lipofectamine RNAiMAX with KAT2A siRNA (Thermo 4390824, s5658) and negative control siRNA (Thermo 4390843).

Cell pellets were lysed with 1% NP-40 lysis buffer (1% NP-40, 25 mM Tris pH 8, 150 mM NaCl, 10% glycerol) supplemented with a protease inhibitor cocktail (Sigma) by incubating them on ice and vortexing the samples every 10 min for 30 min. Supernatant was clarified at 17 000*g* for 20 min at 4 °C. The remaining cell pellet was gently rinsed with 1% NP-40 lysis buffer and further lysed using 4% SDS lysis buffer (4% SDS, 50 mM NaCl, 50 mM triethanolamine) supplemented with a protease inhibitor cocktail (Sigma) and Pierce universal nuclease. Samples were incubated at room temperature and subjected to bath sonication for 5 min, then spun down at 17 000*g* for 10 min at room temperature.

Then, 6× SDS loading dye was added to each sample, and they were heat denatured at 95 °C for 10 min before loading onto a SDS-PAGE gel for analysis. Lysine acetylation, succinylation, and malonylation were determined by Western blot using PTM Biolabs PTM-101, 401, and 901 antibodies, respectively. Membranes were stained with Coomassie Blue to assess loading. Quantification of histone acetylation, succinylation, and malonylation was done using ImageJ. Significance was determined using one-way ANOVA analysis in Prism.

### In Vitro Histone Reaction with KAT2A for Mass Spectrometry (MS) Analysis

Commercially purchased calf thymus histone (15 ug) was added to the reaction buffer (25 mM NH_4_HCO_3_) with or without the 500 nM KAT2A catalytic domain (497–662). Then, 100 μM acetyl-, succinyl-, or malonyl CoA was added, and samples were incubated at 37 °C for 30 min. Control samples containing only calf thymus histone in reaction buffer were also incubated during this time. Each reaction was performed in triplicate.

### In Solution Digestion of Histone Samples

In solution digestion for each sample was performed with an S-Trap microspin column (ProtiFi, Huntington, NY, USA) following an S-Trap protocol as described previously^18,19^ with slight modifications. Thirty micrograms of proteins in 25 μL of buffer containing 50 mM TEAB (pH 8.5), 6 M urea, 2 M thiourea, and 1% SDS was reduced with 15 mM dithiothreitol (DTT) for 1 h at 34 °C, alkylated with 50 mM iodoacetamide for 1 h in the dark, and then quenched with a final concentration of 25 mM DTT. After quenching, 12% phosphoric acid was added to each sample for a final concentration of 1.2%, followed by 1:7 dilution (v/v) with 90% methanol and 0.1 M TEAB (pH 8.5). Each of the resulting samples was then placed into an S-Trap microspin column and centrifuged at 3000*g* for 30 s. The column was washed three times with 150 μL of 90% methanol and 0.1 M TEAB (pH 8.5). Digestion was performed by adding 25 μL of GluC solution (at 1:10 w/w GluC/proteins) in 0.1 M phosphate buffer (pH 7.7) to the top of the spin column. The spin columns were incubated overnight (16 h) at 37 °C. Following incubation, the digested peptides were eluted off the S-Trap column sequentially with 40 μL each of 50 mM TEAB (pH 8.5) followed by 0.2% formic acid and, finally, 50% acetonitrile and 0.2% formic acid (FA). The three peptide eluates were pooled together and aliquoted into two tubes. Half of them were evaporated to dryness with a Speedvac SC110 (Thermo Savant, Milford, MA) then analyzed by nanoLC-MS/MS directly, while the second half were further digested by chymotrypsin (1:10 w/w, chymotrypsin:proteins) in 50 mM ammonium bicarbonate buffer, then evaporated for nanoLC-MS/MS analysis.

### Histone Analysis by Nano LC/MS/MS

The GluC digests and GluC-chymotrypsin double digests were reconstituted in 0.5% FA for nanoLC-ESI-MS/MS analysis. The analysis was carried out using an Orbitrap Fusion Tribrid (Thermo-Fisher Scientific, San Jose, CA) mass spectrometer equipped with a nanospray Flex Ion Source and coupled with a Dionex UltiMate 3000 RSLCnano system (Thermo, Sunnyvale, CA).^18,20^ The peptide samples (8 μL of single digests and 10 μL of double digests) were injected onto a PepMap C-18 RP nano viper trapping column (5 μm, 100 μm i.d × 20 mm) at a 20 μL/min flow rate for rapid sample loading and then separated on a PepMap C-18 RP nano column (2 μm, 75 μm × 25 cm) at 35 °C. The tryptic peptides were eluted with a 90 min gradient of 5% to 33% ACN in 0.1% FA at 300 nL/min, followed by 8 min ramping to 90% ACN-0.1% FA and a 7 min hold at 90% ACN-0.1% FA. The column was re-equilibrated with 0.1% FA for 25 min prior to the next run. The Orbitrap Fusion is operated in positive ion mode with spray voltage set at 1.6 kV and a source temperature at 275 °C. External calibration for FT, IT, and quadrupole mass analyzers was performed. In a data-dependent acquisition (DDA) analysis, the instrument was operated under a CID-ETD toggle method using the FT mass analyzer in MS scan to select precursor ions followed by 4 s “Top Speed” data-dependent CID ion trap MS/MS scans at 3 *m*/*z* quadrupole isolation for precursor peptides with charged ions and ETD with three to seven charged ions above a threshold ion count of 10 000. The normalized collision energy of CID was set at 30%, while the calibrated parameters were used for ETD MS/MS acquisition. MS survey scans at a resolving power of 120 000 (fwhm at *m*/*z* 200) were used for the mass range of *m*/*z* 350–1600. Dynamic exclusion parameters were set at 45 s of exclusion duration with ±10 ppm exclusion mass width. All data were acquired under Xcalibur 4.3 operation software (Thermo-Fisher Scientific).

### MS Data Analysis

The DDA raw files with CID and ETD toggle MS/MS spectra were subjected to database searches using Proteome Discoverer (PD) 2.4 software (Thermo Fisher Scientific, Bremen, Germany) with the Sequest HT algorithm. The PD 2.4 processing workflow containing an additional node of the Minora Feature Detector for precursor ion-based quantification was used for protein identification and protein/peptide relative quantitation analysis between samples. The database search was conducted against a common contaminant database with added histone sequences, which contains 252 entries. Oxidation on M, deamidation on N and Q, acetylation on K, malonylation on K, and succinylation on K were specified as dynamic modifications. Acetylation, M-loss, and M-loss+acetylation in protein N-terminal were specified as dynamic modifications, and carbamidomethyl on C was specified as a static modification. GluC single digestion and GluC+chymotrypsin double digestion with two miss cleavages allowed were set up for histone analysis of single and double digests, respectively. Only high confidence peptides defined by Sequest HT with a 1% FDR by Percolator were considered for the peptide identification.

Relative quantitation of identified proteins/peptides between the control and treated samples was determined by the Label Free Quantitation (LFQ) workflow in PD 2.4. The precursor abundance intensity for each peptide identified by MS/MS in each sample was automatically determined, and the unique peptides for each protein in each sample were summed and used for calculating the protein abundance with the PD 2.4 software. Proteins/peptides ratios were calculated on the basis of the pairwise ratio for treatment over control samples.

## Results

### Purification and Activity Assessment of KAT2A

**Figure 1:**
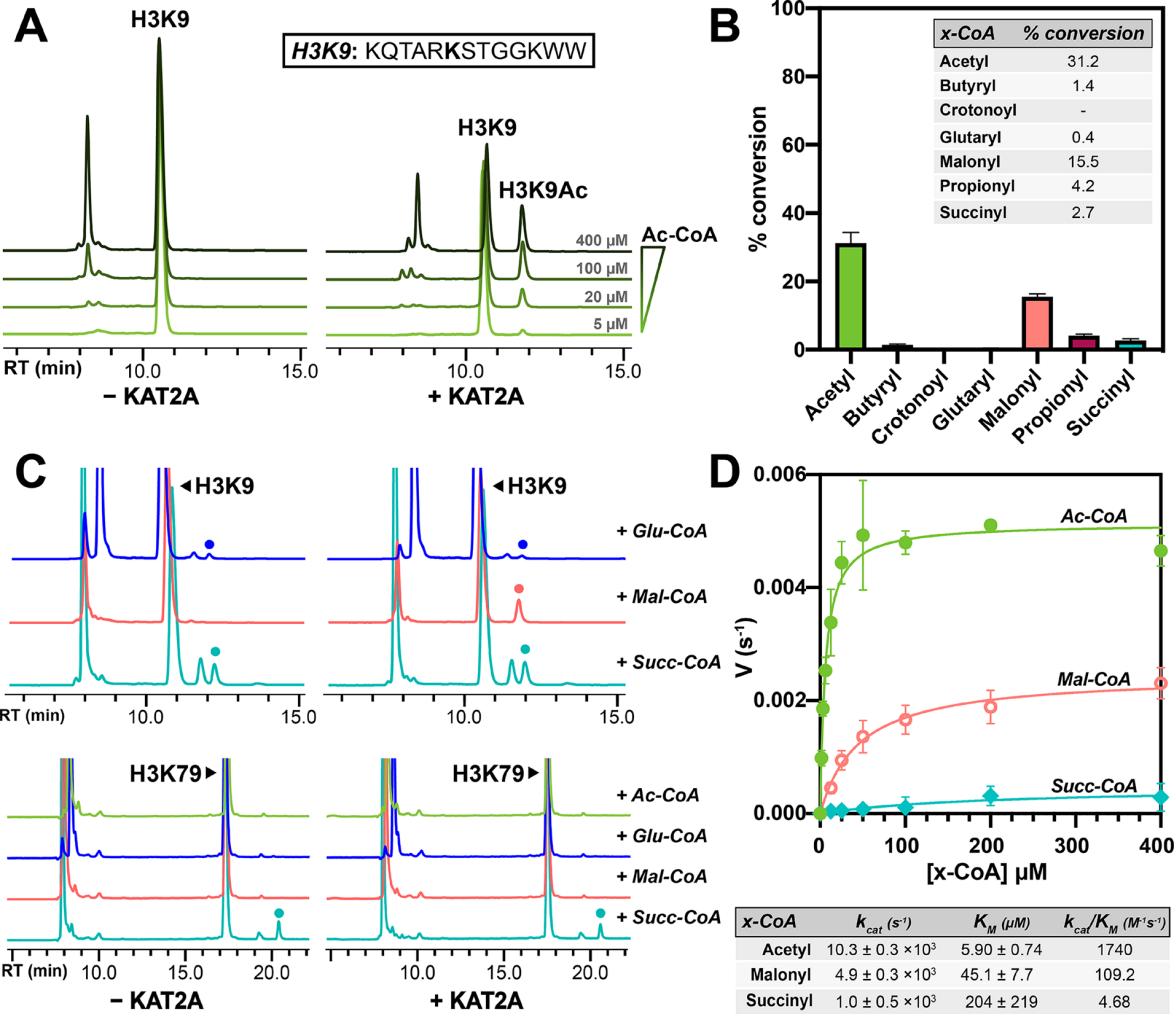
KAT2A catalyzes the transfer of multiple short-chain acyl-CoAs *in vitro*. (A) HPLC trace showing that KAT2A transfers the acetyl group from acetyl-CoA to an H3K9 peptide substrate in an acetyl-CoA concentration-dependent manner. The left panel shows the HPLC traces for reactions without KAT2A, and the right panel shows the HPLC traces for the reaction with KAT2A. The reactions used 5, 20, 100, or 400 μM Ac-CoA and 100 μM H3K9 peptide and 500 nM KAT2A. RT is retention time in minutes. (B) Graph showing % conversion of 100 μM H3K9 peptide to acylated-H3K9 peptide following incubation with 3-fold excess (300 μM) of several short-chain acyl-CoA substrates and 500 nM KAT2A. Background (nonenzymatic) acylation was subtracted. All experiments were performed in duplicate with comparable results each time. (C) HPLC traces of H3K9 and H3K79 peptide following incubation with 4-fold excess (400 μM) acyl-CoA substrates without (left) or with (right) 500 nM KAT2A catalytic domain. RT is retention time in minutes. All experiments were performed in triplicate with comparable results each time. (D) Reaction rate (with the H3K9 peptide) per minute as a function of acyl-CoA concentration and corresponding kinetic parameters following Michaelis–Menten nonlinear regression fitting. Each condition was repeated in triplicate.

The catalytic domain of human KAT2A was cloned and purified as described in the Materials and Methods. To assess whether the purified enzyme was active, we performed an *in vitro* acetylation activity assay using an H3K9 peptide and acetyl-CoA as substrates, analyzing the results via HPLC (Figure 1A). A single peak is observed for the acetylated peptide product at all tested CoA concentrations. Therefore, KAT2A was able to acetylate H3K9 in an acetyl-CoA concentration-dependent manner, confirming that our protein was active.

### Characterization of KAT2A Activity with Multiple Short-Chain Acyl-CoA Substrates

Given the reported glutaryl- and succinyltransferase activity for KAT2A,^15,16^ we screened several short chain acyl-CoAs to determine whether these could behave as efficient substrates (Figure 1B). Surprisingly, while we did see succinyl H3K9 peptide formation, the succinylation without KAT2A occurred almost as efficiently (Figure 1C). Similarly, with glutaryl-CoA, we saw essentially no enzymatic glutaryl H3K9 peptide product formation as the traces with and without KAT2A were very similar (Figure 1C). Even propionylation activity was very minimal (∼4% conversation as compared to ∼30% for acetylation, Figure 1B) despite only a single carbon difference compared to acetyl-CoA. However, interestingly, we found that malonylation of H3K9 by KAT2A is rather efficient (∼15% conversion). The HPLC traces for these reactions are shown in Figure 1C, where a single peak was observed for enzymatic malonylation of H3K9, but multiple peaks were observed with or without KAT2A in the glutarylation and succinylation reactions. The formation of multiple peaks is likely due to an additional lysine residue (K4) in the H3K9 peptide substrate, which is not a substrate in the KAT2A-catalyzed reaction but susceptible to nonenzymatic acylation by reactive CoA species.

To rule out potential sequence selectivity for KAT2A catalyzed succinylation or glutarylation. We tested the succinyltransferase activity of KAT2A using an H3K79 peptide, which was reported to be succinylated by KAT2A. However, we were unable to observe any enzymatic succinylation of the H3K79 substrate as essentially all of the succinylation on H3K79 appeared to be nonenzymatic. Similarly, we were unable to observe any transferase activity on H3K79 using acetyl-, malonyl-, or glutaryl-CoA as a substrate (Figure 1C). The nonenzymatic acylations are consistent with previous reports.^21,22^ Interestingly, the nonenzymatic succinylation and glutarylation on the H3K79 peptide were lower than those on the H3K9 peptide. The H3K9 peptide (KQTARKSTGGKWW) is overall positively charged, while the H3K79 peptide (EIAQDFKTDLRFQWW) is not positively charged, containing one glutamate and two aspartate residues. The positively charged H3K9 peptide may bind to the negatively charged CoA better, thus promoting nonenzymatic acylation.

To further probe the observed CoA specificity of KAT2A, we performed a Michaelis–Menten kinetics experiment using acetyl-, succinyl-, and malonyl-CoA as substrates (Figure 1D). Using the data, we were able to extrapolate *K*_M_ values for the H3K9 peptide and all three acyl-CoAs. While the determined *K*_M_ for acetyl-CoA was a reasonable value of 5.90 μM, the *K*_M_ values for succinyl- and malonyl-CoA were much higher, 45.1 μM and 204 μM, respectively. Further, the conversion rate to succinylated peptide was very low, leading to a very large standard error.

### Characterization of KAT2A Activity on Histone Protein Substrates

**Figure 2:**
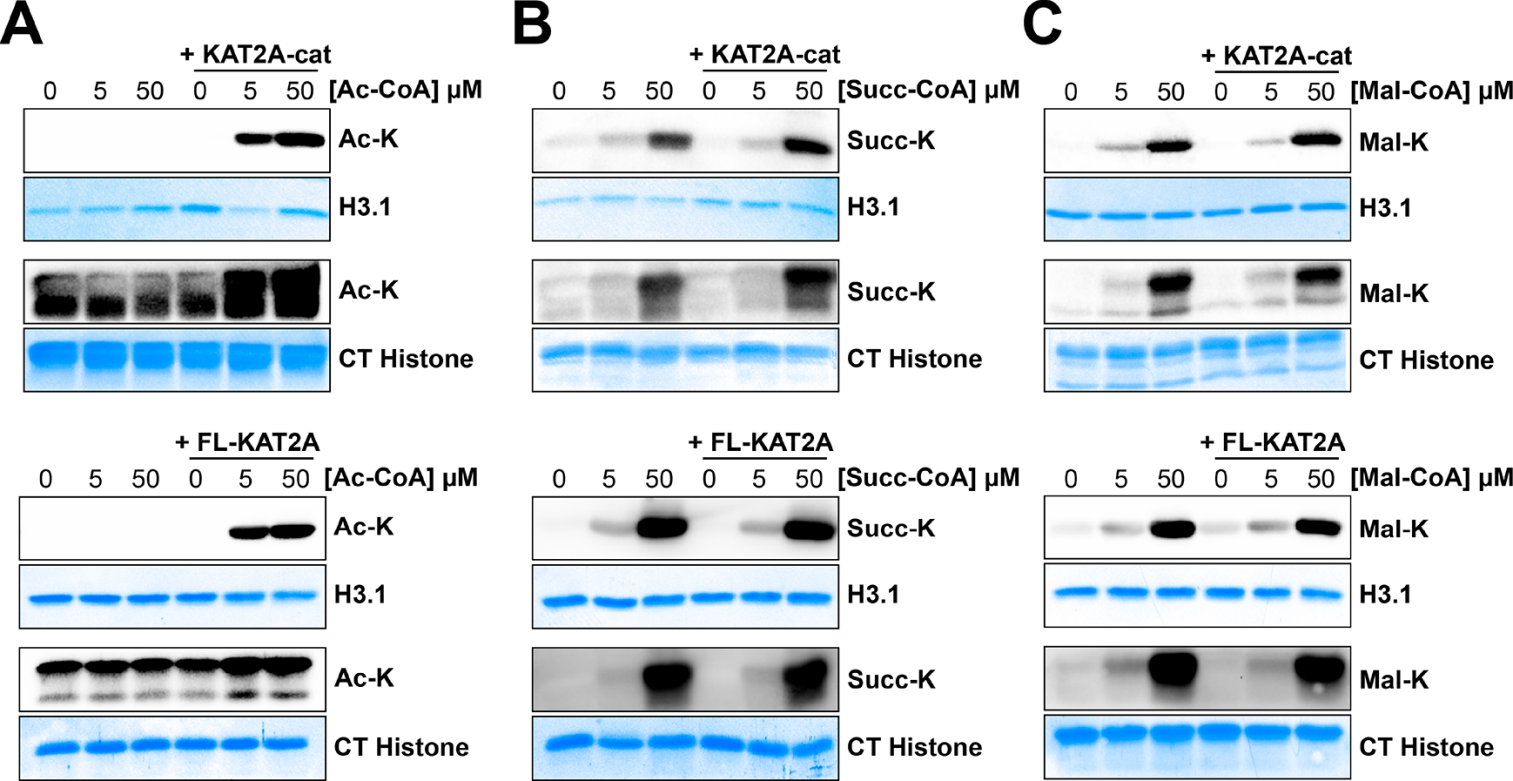
KAT2A primarily catalyzes acetylation on recombinant histone substrates. Western blots of (A) acetyl-, (B) succinyl-, and (C) malonyl-lysine following *in vitro* activity assay with 0, 5, or 50 μM acyl-CoA, KAT2A-catalytic domain (top) or full-length KAT2A (bottom), and histone H3.1 or calf thymus (CT) histone. Membranes were stained with Coomassie Blue for determining protein loading. All experiments were performed in triplicate with reproducible results each time.

Our above *in vitro* data suggest that acetylation is the major activity for KAT2A. However, these results were based on modification of an H3K9 peptide, which only covers one possible sequence and may not be the ideal substrate for KAT2A-catalyzed transfer. Therefore, we performed a similar experiment using recombinant histone substrates to further understand the activity of KAT2A. For this assay, the recombinant KAT2A catalytic domain or commercially purchased FL-KAT2A was incubated with recombinant H3.1 and histone isolated from calf thymus in the presence of 5 or 50 μM of acetyl-, succinyl-, or malonyl-CoA. Reactions were heat denatured and analyzed via Western blot with the corresponding acyl-lysine antibodies. A robust increase in acetylation was observed for histone samples with KAT2A and acetyl-CoA (Figure 2A). In contrast, with succinyl-CoA or malonyl- CoA, the acylation levels on both H3.1 and calf thymus histone samples were similar compared to those with and without KAT2A, especially at a 5 μM acyl-CoA concentration (Figure 2B and C). Thus, even with full-length histones, acetylation still appears to be the major activity for KAT2A.

### Determination of Histone Modification by KAT2A Using Mass Spectrometry

**Figure 3:**
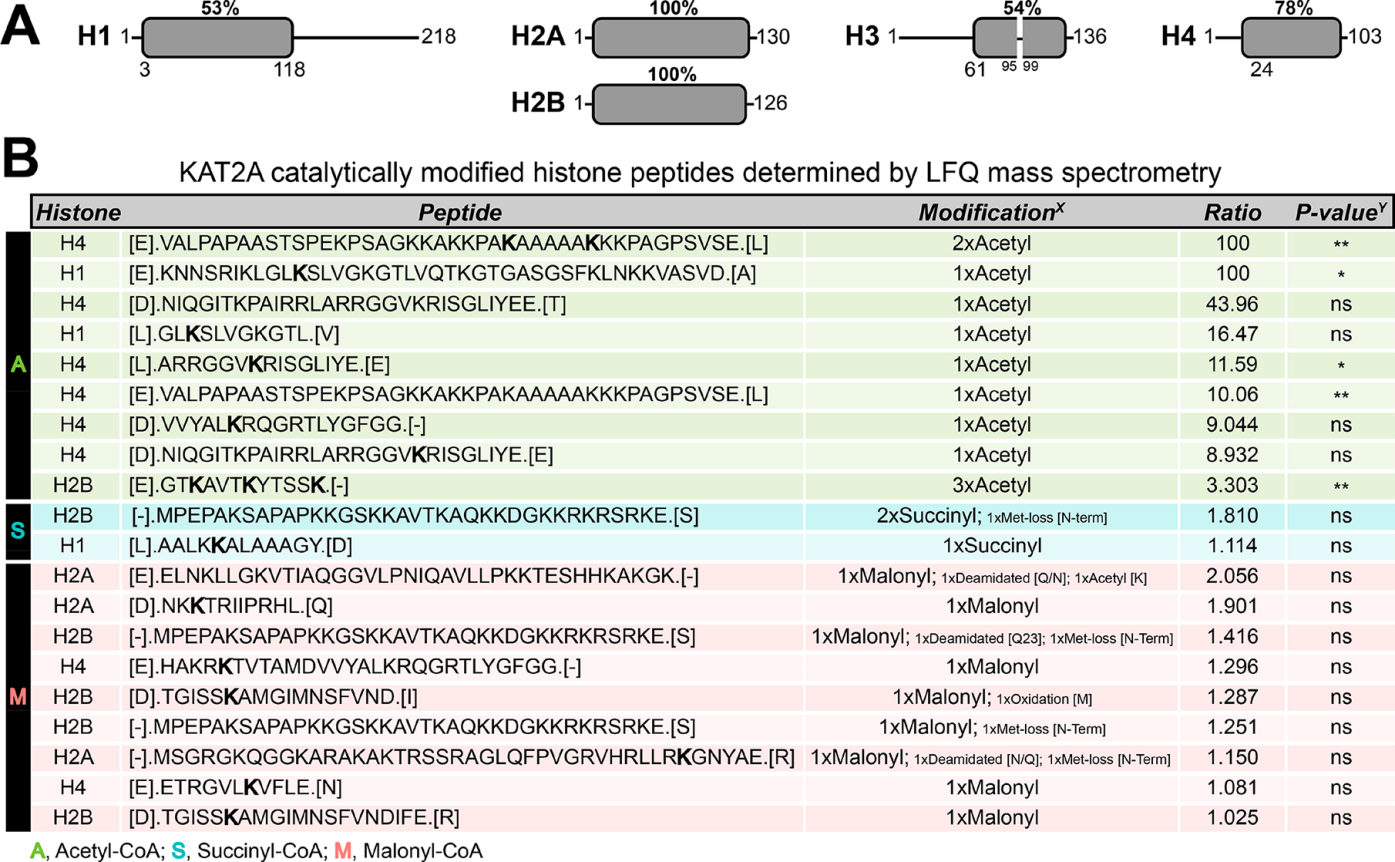
KAT2A robustly catalyzes histone acetylation as determined by label-free quantification mass spectrometry. (A) Histone coverage in the LFQ MS experiment. Coverage region is indicated in gray with the percent of overall histone shown above. (B) Table summarizing LFQ MS results for KAT2A modified histone peptides with modified lysines bolded. Ratios represent fold enrichment in samples containing KAT2A vs control samples without KAT2A. All peptides with ratios > 1 are shown for reactions with succinyl- and malonyl-CoA. Peptides with ratios > 3 are shown for reactions with acetyl-CoA. Additional nonsignificant (ns) acetyl-lysine peptides with ratios > 1 were found and are available in the Supporting Information. ^X^All modifications are found on lysine if the amino acid is not indicated. For peptides where modified lysine is not bolded, corresponding masses were found, but the modification site could not be identified specifically. ^Y^*P* values were determined using unpaired *t* test analysis in Prism. *P* values of >0.05 (ns), ≤0.05 (*), and ≤0.01 (**). All peptides with significant abundance are shown in the table.

To further confirm the KAT2A-catalyzed histone acylation results obtained with WB, we used label-free quantification (LFQ) mass spectrometry (MS) to quantify the acylation levels on calf-thymus histones after the in vitro acylation reaction with and without KAT2A. Calf thymus histone was incubated with 50 μM acetyl-, succinyl-, or malonyl-CoA in the absence or presence of the KAT2A catalytic domain. The histones were digested with GluC and/or chymotrypsin, and the resulting peptides were identified by MS. As expected, several histone peptides were identified with acetyl-, succinyl-, or malonyl modifications. Acyl peptide abundance ratios were determined for samples with KAT2A vs without KAT2A by comparing the median abundance value in samples with or without KAT2A (Figure 3). Each reaction was done in triplicate with identical histone input, so median abundance provides a representative value for determining peptide ratios. The determined *p* values consider raw peptide abundance for all replicates. Following digestion, at least 50% sequence coverage was achieved for each histone (Figure 3A). Compared to samples without KAT2A, KAT2A dramatically increased the acetylation level on multiple histone peptides. In contrast, succinylated and malonylated peptides were found in similar abundance between samples with or without KAT2A. A few peptides showed increased succinyl- or malonyl-lysine with KAT2A, but the determined abundance ratios were low (<2.5-fold enrichment) and not significant (*p* value >0.05) by *t* test analysis. Thus, acetylation appears to be the only significant activity of KAT2A on histone proteins *in vitro*.

### Effect of Transient Knockdown of KAT2A in HEK293T Cells

**Figure 4:**
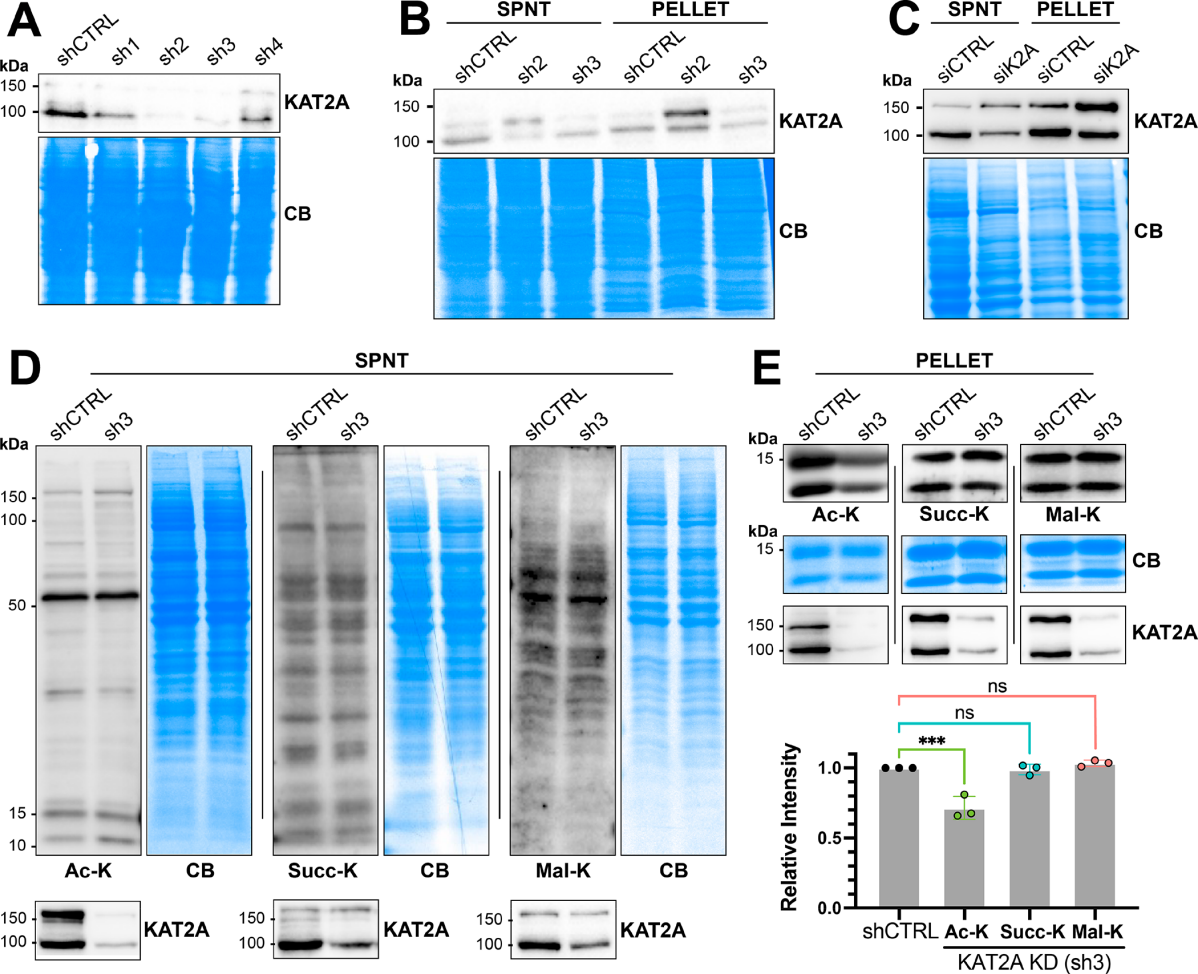
Transient knockdown of KAT2A leads to a decrease in histone acetylation. (A) Western blot of HEK293T lysate supernatant confirming that KAT2A is knocked down relative to the nontargeting control. (B) Western blot showing that transient shRNA knockdown leads to expression of a compensatory isoform in both the supernatant (spnt) and pellet fractions with sh2. (C) Western blot showing that siRNA knockdown of KAT2A leads to expression of a higher molecular weight compensatory isoform. (D) Western blot showing that global lysine acetylation, succinylation, and malonylation of 293T supernatant does not change with KAT2A knockdown. (E) Western blot probing lysine acetylation, succinylation, and malonylation of the histone portion of lysed 293T cell pellet. Quantification of lysine modification relative to the control for three independent replicates is shown in the bar graph below. *P* value for lysine acetylation decrease is 0.0002. All experiments were performed in triplicate with similar results each time. Coomassie Blue (CB) staining of the membranes shows equal loading for all experiments.

Thus far, all of our experiments were performed with KAT2A in an *in vitro* system. Therefore, to further characterize this activity in a cellular system, KAT2A was transiently knocked down via shRNA in HEK293T cells (Figure 4A). The shRNA’s sh2 and sh3 gave the strongest knockdown level (Figure 4A).

However, attempts to generate a stable KAT2A KD cell line led to the rescue of KAT2A after a single passage. Similarly, transient knockdown of sh2 showed a compensatory KAT2A isoform in the pellet fraction (Figure 4B), and siRNA knockdown shows the compensatory isoform in both fractions (Figure 4C). Thus, all experiments probing changes to global and histone lysine modification were performed by transient knockdown with sh3, which did not show the appearance of a compensatory isoform following transient knockdown for 72 h. Cells were collected 72 h post-transfection and lysed using standard 1% NP-40 lysis buffer to isolate the soluble cytosolic fraction. The insoluble fraction was further lysed using 4% SDS lysis buffer to probe changes in histone modification.

Following KAT2A knockdown and blotting for lysine modifications in the supernatant fraction, some minor changes in acetylation were observed, while no obvious changes in succinylation or malonylation were observed (Figure 4D). The minor changes to the lysine acetylation blot in the supernatant fraction suggest that there are likely nonhistone KAT2A acetylation substrates. Knockdown of KAT2A led to obvious decreases in the acetylation levels in the pellet fraction, which contains histones. The sizes of the acetylation bands were consistent with those of histones, suggesting that a loss of KAT2A led to a significant decrease in histone acetylation (Figure 4E). In contrast, we saw no obvious changes in histone lysine succinylation or malonylation levels upon KAT2A knockdown, consistent with our observation that KAT2A did not significantly increase histone succinylation or malonylation *in vitro* (Figure 2 and 3; Figure 4E). Therefore, the major physiological activity for KAT2A in HEK293T cells appears to be histone acetylation.

## Discussion

Lysine acetylation has been widely recognized as an important PTM that regulates many biological pathways.^23^ Accordingly, writers, readers, and erasers of acetyl-lysine code have been well-known. Recent identification of many other acyl lysine modifications has made it necessary to identify their writers, readers, and erasers in order to fully understand the physiological significance of the these new acyl lysine modifications.^23^

In terms of writers, it has been reported that several lysine acetyltransferases can also transfer other acyl groups to protein lysine residues. For example, p300 and CBP catalyze propionylation, butyrylation, and crotonylation of histones and p53.^2,24^ Similarly, human P/CAF was reported to catalyze propionylation of histone H3 peptides.^25^ However, these acyl groups are chemically more similar to acetyl, and thus it is probably not surprising that the acetyltransferases would be able to accept them as substrates with lower catalytic efficiencies. The reports that KAT2A can catalyze glutarylation and succinylation are more surprising, as these acyl groups are negatively charged and structurally more divergent from acetyl. In addition, for lysine succinylation, nonenzymatic reactions with succinyl-CoA are well-established, and thus writers of succinylation may be unnecessary, especially in environments where succinyl-CoA may exist in high local concentrations.^26,27^

These considerations prompted us to carefully examine the ability of KAT2A to use various short-chain acyl-CoA molecules as substrates. Our study shows that acetylation is the most efficient activity of KAT2A. While malonylation and propionylation were also observed in vitro using an H3K9 peptide, these activities are much lower compared to those of acetylation. In contrast, we did not see obvious succinylation activity, while nonenzymatic succinylation was readily detected. Similarly, we did not observe any efficient glutarylation activity with KAT2A *in vitro* (Figure 1B,C). In HEK293T cells, we showed that KAT2A knockdown reduces global histone acetylation level, but not histone malonylation or succinylation levels (Figure 4E). Our results suggest that KAT2A’s major physiological activity is acetylation. Our results are consistent with a report from 2016 which showed that KAT2A catalyzes propionylation and butyrylation, but with much lower efficiency compared to acetylation.^28^ Structural data (e.g., PDB 5h84) suggest that, while the active site of KAT2A can accommodate the longer butyryl chain, steric clashes between the active site and the butyryl group occurs, preventing efficient transfer.

Our data show that, overall, KAT2A is mainly an acetyltransferase and its succinyltransferase/glutaryltransferase activity is very weak. While we could not rule out that the succinylation/glutarylation of certain specific lysine residues on certain proteins could be controlled by KAT2A, based on our data presented here, we believe that the reported histone succinylation reaction catalyzed by KAT2A comes from nonenzymatic acylation. Our results suggest that any claim about KAT2A catalyzing succinylation/glutarylation must be more carefully scrutinized.

**Figure 5:**
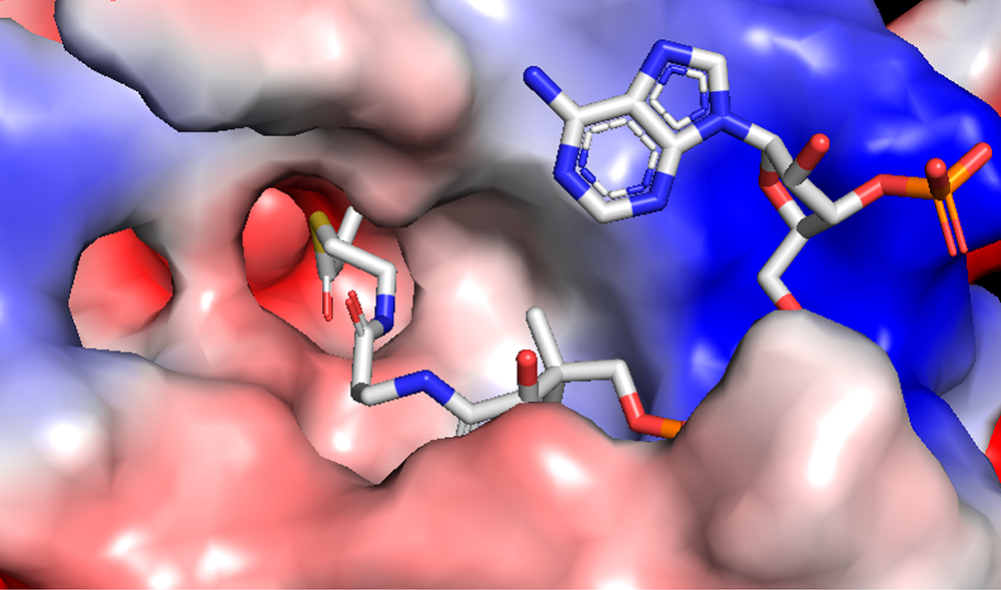
Possible explanation for the preference of malonyl-CoA over succinyl-CoA by KAT2A. The structure of KAT2A in complex with propionyl-CoA (PDB 5h84) is used for this analysis. KAT2A’s surface contact potential map (generated using PyMOL) is shown, with the blue color indicating positive potential, the red color indicating negative potential, and the white color indicating a neutral surface. The propionyl-CoA molecule is shown in stick representation. The propionyl group is next to a negative surface of KAT2A, to which malonyl and succinyl-CoA would also be adjacent if they are bound to KAT2A. The longer succinyl-CoA would present the negatively charged carboxylate closer to the negative surface of KAT2A, causing an unfavorable interaction and explaining why succinyl-CoA would be a worse substrate than malonyl-CoA.

Interestingly, our *in vitro* data using an H3K9 peptide suggest that KAT2A could catalyze lysine malonylation rather efficiently. Using the published structure of KAT2A in complex with propionyl-CoA bound,^28^ we rationalized the preference for malonyl-CoA over succinyl-CoA by KAT2A (Figure 5). The propionyl group is next to a negative surface of KAT2A (formed by several backbone amide carbonyl groups), to which malonyl and succinyl-CoA would also be adjacent if they are bound to KAT2A. The longer succinyl-CoA would present the negatively charged carboxylate closer to the negative surface of KAT2A, making the interaction less favorable. With the shorter malonyl-CoA, the repulsion would be less, thus explaining the preference for malonyl-CoA over succinyl-CoA. The repulsion could also explain why acetyl-CoA is a better substrate than malonyl-CoA.

While this malonylation activity was not observed with full length histones as substrates, the efficient malonylation activity of KAT2A on a peptide suggests that it may be productive to examine potential malonylation substrates of KAT2A. Malonylation has been reported to occur on many proteins.^4,29,30^ It may be interesting to explore whether some of these malonylated proteins are regulated by KAT2A in future studies.

## Conclusions

In conclusion, our data showed that KAT2A’s major activity *in vitro* and in HEK293T cells is acetylation. In contrast to previous reports, we found that the succinylation and glutarylation activities of KAT2A are hardly detectable *in vitro* and dominated by nonenzymatic acylation. These results suggest that the claims about KAT2A being a succinyltransferase or glutaryltransferase are unlikely to be true and need to be more carefully scrutinized and validated. Further, KAT2A’s malonylation activity on an H3K9 peptide is much more efficient than succinylation or glutarylation and would be interesting to follow up on in the the future.

## Supporting Information Available

Identified histone peptides and raw abundance values for each digestion condition (GluC and/or chymotrypsin) and CoA (acetyl-/succinyl-/malonyl-). Peptides with appropriate modifications and abundance ratios >1 are highlighted (XLSX)

## Accession Codes

Human KAT2A (Q92830), Bovine H1 (G3N131), Bovine H2A (P0C0S9), Bovine H2B (P62808), Bovine H3 (P68432), Bovine H4 (P62803)
